# Radio-frequency ablation-based studies on VX2rabbit models for HCC treatment

**DOI:** 10.1186/s13027-016-0082-9

**Published:** 2016-08-12

**Authors:** Sabrina Bimonte, Maddalena Leongito, Mauro Piccirillo, Cristina de Angelis, Claudia Pivonello, Vincenza Granata, Francesco Izzo

**Affiliations:** 1 Division of Abdominal Surgical Oncology, Hepatobiliary Unit, Istituto Nazionale per lo studio e la cura dei Tumori “Fondazione G. Pascale”, - IRCCS, Via Mariano Semmola, 80131 Naples, Italy; 2 I.O.S. & Coleman Srl, Naples, Italy; 3 Dipartimento di Medicina Clinica e Chirurgia, Sezione di Endocrinologia, Università di Napoli Federico II, Naples, Italy

**Keywords:** Hepatocellular carcinoma, RFA, Radiofrequency ablation, Vx2 tumors, Residual tumor

## Abstract

Hepatocellular carcinoma (HCC) is the fifth most frequent cancer worldwide with high morbidity, mortality and increasing incidence. It is of note that the main curative therapies for HCC are hepatic resection and transplantation although the majority of patients at the time of presentation are not eligible for resection or orthotopic liver transplantation (OLT) due to the underlying cirrhosis. Currently, a variety of loco-regional therapies, including radiofrequency ablation (RFA), percutaneous ethanol injection (PEI), microwave coagulation therapy (MCT), transarterial chemoembolization (TACE) and others, have been developed as alternative treatment options for HCC. Among these techniques, RFA is currently the most widely used treatment, due to its several advantages, such as safety and efficacy. To date, the effectiveness of RFA for HCC is reduced by the presence of residual tumor as a consequence of insufficient treatment. In order to ameliorate the effects of RFA on HCC, several in vivo studies, have been performed on its application as single or in combination treatment with drugs or others loco-regional therapies, by using rabbit VX2 liver model. This represents an ideal model of liver cancers and is widely used for imaging and other experimental studies due to the rapid growth of these tumors and their similarity to human hepatocellular carcinoma. In order to elucidate the therapeutic potential of RFA with adjuvant treatments for HCC, we reviewed the latest findings on the RFA-based studies in rabbit VX2 hepatocarcinoma models.

## Background

HCC is still one of the most important diseases for health care systems due to its high mortality, morbidity, and increasing incidence worldwide [1, 2]. It is of note that the main curative therapies for HCC are hepatic resection and transplantation although the majority of patients at the time of presentation are not eligible for resection or OLT due to the underlying cirrhosis. Difficulties in surgical resection may be associated to site, size, and number of tumors, vascular and extra hepatic involvement as well as the general condition and liver function of the patients [3–6]. Only about 20 % of HCC cases are classified as resectable [7]. Moreover, the liver is considered a site of metastasis from other solid cancers [8–12]. To date, a variety of loco-regional therapies, RFA, PEI, MCT, TACE and others, have been developed as alternative treatment options for HCC due to its benefits, such as safety, minimal invasiveness, and efficacy [7]. It has been demonstrated that the effectiveness of RFA for HCC was reduced by the presence of residual tumor and local recurrence after treatment, probably due to the location of tumor around the intrahepatic vasculature [13] or to the diameter of tumor. Several studies showed that, residual tumor progression after insufficient RFA could be associated to different reasons and molecular mechanisms [14–18] in particular to the inflammation process which is involved in tumor progression of different types of cancer [19, 20]. The inflammation is induced by thermal destruction of liver carcinoma after RFA at the target sites, leading to progression of HCC tumor [20].

In order to ameliorate the effects of RFA on HCC and to bypass the problem of residual tumor, several in vivo studies have been performed on its application as single or in combination treatment with drugs or other loco-regional therapies, by using rabbit VX2 liver carcinoma model [18, 21–24].

The rabbit VX2 tumor model is widely used in experimental oncology. It is classified as leporine anaplastic squamous cell carcinoma being characterized by rapid growth, hypervascularity and easy propagation in the skeletal muscle [25–28]. This model has been applied to various types of cancer [29–38], and recently it has been used in doxorubicin interventional chemotherapy of renal carcinoma [39]. The transplantation of the VX2 cells can be achieved either by injecting the tumor cell suspensions or by implanting solid tumor pieces (fresh o frozen) as previously described [40].

This represents an ideal model of liver cancers due to the rapid growth of VX2 tumors and their similarity to human hepatocellular carcinoma. Here we reviewed the latest findings on the RFA based studies in rabbit VX2 hepatocarcinoma models, with the aim of elucidating the therapeutic potential of RFA with adjuvant treatments for HCC.

### Generation of VX2 model for liver tumor treatment: an overview of loco regional therapy-based studies

**Fig. 1:**
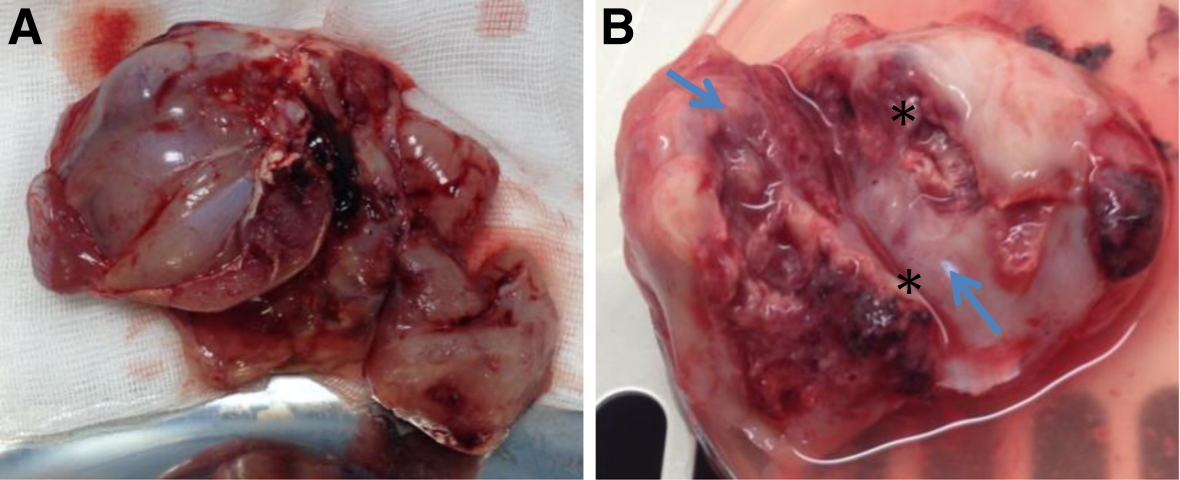
VX2 tumor liver development. **a** Picture reveals resected hepatic tumor after 4 week from VX2 tumor pieces implant, **b** Tumor bisected shows necrotic core (*asterisk*) and peripheral viable tumor (*arrows*)

Several studies demonstrated that intrahepatic implantation of solid tumor fragments is more successfully that the injection of a cell suspension [41–43], although a sonography implantation of liver tumors achieved a good success rate [44]. The protocol for liver implantation has been detailed described by Parvivian et al. [40]. It is of note that the preferred implantation site for VX2 tumors is the left lateral hepatic lobe due to a more favorable angle of the feeding artery for later angiographic catheterization. Our group and other researchers demonstrated that the tumors developed after 2–4 weeks form tissue implantation with nodules of 2-3 cm [38] (Fig. 1).

It also has been reported that the application of ultrasonography, is useful to following the solid tumor growth and to detect the presence of necrosis [44]. The use of non-necrotic tumors allows optimizing the evaluation of tumor response to loco-regional therapy experiments. It is largely provided that loco-regional therapies, including RFA and TACE, play a major role in the clinical management of hepatocellular carcinoma [45]. Regarding pre-clinical studies, many evidences reported the safety and the efficacy of different loco-regional treatments in rabbit VX2 liver tumor model, especially in combination with adjuvant substances. Gholamrezanezhad et al.*,* evaluated the pharmacokinetic profile (PK) and embolization effect of 70–150-μm doxorubicin eluting beads (DEBs) following intra-arterial injection, in the rabbit liver VX2 tumor model [46]. In VX2 model of liver metastases, it has been reported that HepaSphere and DEBS microspheres loaded with irinotecan, caused significant necrosis of tumor nodules [47]. Xia et al.*,* showed that intra-arterial interleukin-12 gene delivery combined with chemoembolization, had a potent anti-tumor effect in a VX2 rabbit HCC [48]. In another study performed on the rabbit VX2 liver tumor model, was demonstrated that the application of Electroporation-Mediated Transcatheter Arterial Chemoembolization (E-TACE) increased liver tumor chemotherapeutic uptake following targeted transcatheter infusion [49]. In addition, Deng et al., showed that TACE with arterial administration of Endostar (an antiangiogenic agent) inhibited the angiogenesis biomarkers associated with TACE in a rabbit model bearing VX2 liver tumor [50]. Potentiated anti-tumor effects on liver cancer, were also observed in a similar study with chloroquine and TACE. The authors showed that chloroquine, which is a traditional drug used for treatment of malaria [51], promoted the anticancer effect of TACE in a rabbit VX2 liver tumor model. Other studies proved that single-bolus regional chemotherapy with doxorubicin, had limited anti-tumor effects when compared with TACE in a rabbit VX2 liver tumor model [52]. Very recently, it has been reported that the elaborate integration of TACE with nanoparticle-enhanced High-intensity focused ultrasound (HIFU) cancer surgery, applied in VX2 liver tumor model, could efficiently enhance the HCC cancer treatment outcome, representing a new and efficient therapeutic protocol/modality for clinic cancer treatment [53]. Another research demonstrated that Transarterial oily chemoembolization (TOCE), one of the most effective approaches for the treatment of patients with HCC, who are not suitable for surgical therapy [54], combined with Lidamycin (LDM) [55], shows potent therapeutic efficacy in VX2 rabbit liver tumor model. Synergistic effects of RFA and toll like receptor 9 (TRL9) stimulation result in a potentiated antitumor T cell response and cytotoxicity in the VX2 hepatoma tumor [23].

Taken together all these different data, summarized in Table 1, suggest that these combined treatments may have potential synergistic effects on liver cancer.

**Table 1:** Effects of Loco-regional treatments on tumor growth in VX2 rabbit model of liver cancer

Animal model	Treatment	Effects on tumor	Reference
Rabbit Vx2 liver tumor model	TACE, DEBs	*Tumor growth reduction*	[46]
Rabbit VX2 liver metastis model	HepaSphere and DC Bead microspheres loaded with	*Enhanced Tumor necrosis*	[47]
Rabbit VX2 HCC model	IL12, TACE	*Tumor growth reduction*	[48]
Rabbit VX2 HCC model	Endostar, TACE	*Tumor growth reduction*	[50]
Rabbit VX2 liver model	Chloroquine, TACE	*Tumor growth reduction*	[51]
Rabbit VX2 liver model	TACE, HIFU	*Tumor growth reduction*	[53]
Rabbit VX2 liver tumor model	LDM, TOCE	*Tumor growth reduction*	[54]
Rabbit VX2 HCC model	TRL9, RFA	*Tumor growth reduction*	[23]

Abbreviations: *TACE* transcatheter arterial chemoembolization, *IL-12* interleukin-12, *LDM* Lidamycin, *TOCE* transarterial oily chemoembolization, *HIFU* high-intensity focused ultrasound, *TRL9* toll like receptor 9

### Radio-frequency ablation of VX2 rabbits model for HCC treatment: experimental studies

RFA is considered one of the standard procedures for local tumor treatment such as prostate [56, 57], kidney [56], bone [58, 59], brain [60, 61], lung [62, 63] and liver tumors [45, 64–67]. RFA is commonly used for the treatment of early HCC, although recently the technique has been improved to bypass the problem of burden for patients and operators [68]. RFA is classified as thermal technique since induces cell tumor destruction by heating tumor tissue to high temperatures [69, 70]. It is important to underline that the effectiveness of RFA for HCC is reduced by the presence of residual tumor as a consequence of insufficient treatment. It is very difficult to avoid the residual tumor due to several causes such as the liver’s anatomy, the mechanisms of RFA, the pathological characteristics of HCC and the inflammation. It has been demonstrated that despite it is possible to set the target temperature around 105–115 °C during RFA, only the tissues surrounding the electrodes can reach that temperature due to “heat sink” effect of blood large vessels near the tumor [71].

Many evidences have demonstrated a rapid progression of residual HCC after RFA [72, 73]. In addition, this tumor becoming very aggressive, switches to sarcoma [74, 75] thus leading to bad prognosis for patients.

In order to ameliorate the effects of RFA on HCC and to clarify the underlying mechanisms of rapid progression of residual tumor after insufficient RFA, several in vivo studies, have been performed using rabbit VX2 hepatocarcinoma model. The study conducted by Ke et al., was designed to prove whether low temperature of RFA at the target sites, and could facilitate progression of residual hepatic VX2 carcinoma trying to dissect the underlying mechanisms. The VX2 nodules were transplanted into the liver rather than derived from the liver itself, in order to reduce the feeding artery and the heat sink effect. The residual VX2 hepatoma model in rabbits was established by using RFA at different temperatures (55, 70 and 85 °C). Actually, it is of note that different molecular factors are involved in HCC progression and metastasis, such as IL-6, PCNA, MMP-9, VEGF, HGF [76–78]. The authors demonstrated that residual hepatic VX2carcinoma facilitated its progression through inducing over expression of several molecular factors, such as PCNA, MMP-9, VEGF, HGF and IL-6 [18].

In another research, was proved that the inflammation induced by RFA at the target sites, facilitated the progression of residual HCC tumor [21]. The authors generated the orthotropic VX2 rabbit HCC model with two hepatic tumors in different lobes and treated the animals with different doses of aspirin used as anti-inflammatory drug. Results from this study demonstrated that aspirin inhibited the inflammatory reaction of animals after RFA, suggesting that aspirin could be potentially used as an adjuvant therapy with RFA for treating HCC. Table 2 summirizes  the effects of RFA on the progression of residual HCC in VX2 rabbit model of liver cancer.

**Table 2:** Effects of RFA on the progression of residual HCC in VX2 rabbit model of liver cancer

Animal model	Treatment	Effects on the progression of residual HCC	References
Rabbit VX2 HCC model	RFA at different temperatures (55,70 and 85 °C)	Low temperatures induce rapid progression of residual HCC. Tumor progression is associated to PCNA, MMP-9, and VEGF, HGF and IL-6 overexpression.	[18]
Rabbit VX2 HCC model	Aspirin, RFA	Reduced progression of residual HCC associated to reduced inflammation.	[21]

## Conclusions

Altogether these data suggest that inflammation and low temperature of RFA at the target sites could be important reasons for rapid progression of residual hepatic VX2 carcinoma. Future studies will be needed to validate the therapeutic potential of drugs or other loco-regional techniques as an adjuvant therapy with RFA for treating HCC.

## Abbreviations

(E-TACE), Electroporation-Mediated Transcatheter Arterial Chemoembolization; (HIFU), high-intensity focused ultrasound; DEBs, doxorubicin eluting beads; HCC, hepatocellular carcinoma; HGF, hepatocyte growth factor; IL-6, *Interleukin*-*6*; LDM, lidamycin; MCT, microwave coagulation therapy; MDR, multidrug resistance; MMP-9, matrix metallopeptidase 9; OLT, orthotopic liver transplantation; PCNA, *Proliferating cell nuclear antigen*; PEI, percutaneous ethanol injection; PK, pharmacokinetic profile; RFA, radiofrequency ablation; TACE, transcatheter arterial chemoembolization; TOCE, transarterial oily chemoembolization; TRL9, toll like receptor 9; VEGF, *Vascular endothelial growth factor*
